# Comprehensive Analysis of Necroptosis-Related Long Noncoding RNA Immune Infiltration and Prediction of Prognosis in Patients With Colon Cancer

**DOI:** 10.3389/fmolb.2022.811269

**Published:** 2022-02-14

**Authors:** Li Liu, Liu Huang, Wenzheng Chen, Guoyang Zhang, Yebei Li, Yukang Wu, Jianbo Xiong, Zhigang Jie

**Affiliations:** ^1^ Department of Gastrointestinal Surgery, The First Affiliated Hospital of Nanchang University, Nanchang, China; ^2^ The Second Affiliated Hospital of Nanchang University, Nanchang, China

**Keywords:** necroptosis, lncRNA, colon cancer, prognostic model, bioinformatics

## Abstract

Colon cancer (CC) is one of the most frequent malignancies in the world, with a high rate of morbidity and death. In CC, necroptosis and long noncoding RNA (lncRNAs) are crucial, but the mechanism is not completely clear. The goal of this study was to create a new signature that might predict patient survival and tumor immunity in patients with CC. Expression profiles of necroptosis-related lncRNAs in 473 patients with CC were retrieved from the TCGA database. A consensus clustering analysis based on differentially expressed (DE) genes and a prognostic model based on least absolute shrinkage and selection operator (LASSO) regression analysis were conducted. Clinicopathological correlation analysis, expression difference analysis, PCA, TMB, GO analysis, KEGG enrichment analysis, survival analysis, immune correlation analysis, prediction of clinical therapeutic compounds, and qRT–PCR were also conducted. Fifty-six necroptosis-related lncRNAs were found to be linked to the prognosis, and consensus clustering analysis was performed. There were substantial variations in survival, immune checkpoint expression, clinicopathological correlations, and tumor immunity among the different subgroups. Six lncRNAs were discovered, and patients were split into high-risk and low-risk groups based on a risk score generated using these six lncRNAs. The survival time of low-risk patients was considerably longer than that of high-risk patients, indicating that these lncRNAs are directly associated with survival. The risk score was associated with the tumor stage, infiltration depth, lymph node metastasis, and distant metastasis. After univariate and multivariate Cox regression analysis, the risk score and tumor stage remained significant. Cancer- and metabolism-related pathways were enriched by KEGG analyses. Immune infiltration was shown to differ significantly between high- and low-risk patients in a tumor immunoassay. Eight compounds were screened out, and qRT–PCR confirmed the differential expression of the six lncRNAs. Overall, in CC, necroptosis-related lncRNAs have an important function, and the prognosis of patients with CC can be predicted by these six necroptosis-related lncRNAs. They may be useful in the future for customized cancer therapy.

## Introduction

Colon cancer (CC) is one of the most frequent cancers worldwide. In 2020, it accounted for 6% of all tumors worldwide, and its morbidity and mortality ranked fifth among all cancers (Sung et al., 2021). With the progression of medical technology, its morbidity and mortality have gradually decreased in some developed countries, but it has increased in developing countries in Asia and South America, and its incidence has increased significantly among young people (Brenner et al., 2019; Feletto et al., 2019; Arnold et al., 2020; Islami et al., 2021). Surgery and chemotherapy are the most common treatments for CC, but the effect is usually not satisfactory (DeSantis et al., 20142014). The pathogenesis of CC is not fully clear, and the optimal diagnosis and management of CC is still a source of debate (Arredondo et al., 2020; Biller and Schrag, 2021). In recent years, immunotherapy has emerged as a novel therapeutic option for CC, with promising outcomes (Mlecnik et al., 2020; Trimaglio et al., 2020). However, there are great differences in the curative effect among patients, and many problems need to be solved.

RNA polymerase II transcribes long noncoding RNAs (lncRNAs), described as a set of RNA molecules with a length of more than 200 bp but no protein-coding potential (Wang et al., 2018). Through gene transcription and posttranscriptional modification, lncRNAs play a specific role in carcinogenesis and metastasis (Du et al., 2020; Gao et al., 2020; Liu et al., 2021a). Studies from Balihodzic have shown that the extraction of lncRNAs is related to glucose metabolism tumors (Balihodzic et al., 2021). Chen noted that lncRNAs were associated with tumor autophagy in colorectal cancer (Chen et al., 2021), and lncRNAs were also associated with tumor metabolism and tumor metastasis (Xu et al., 2021). Barik also summarized the significance and clinical application of lncRNAs in tumor drug resistance (Barik et al., 2021); for example, MALAT1 has obvious significance in the diagnosis, prognosis, and treatment of many kinds of tumors (Goyal et al., 2021). At the same time, lncRNAs may inhibit the immune microenvironment (Pi et al., 2021). Although lncRNAs play a crucial role in the onset and progression of tumors, the current research is still not completely clear.

Schweichel and Merker divided cell death into three types according to morphology in 1973 (Schweichel and Merker, 1973), and later researchers named them apoptosis, autophagic cell death, and necrosis (Kroemer et al., 2009). With additional research, another mechanism of cell death, necroptosis (programmed necrosis), was discovered in 1988 as a type of programmed death and was named in 2005 (Laster et al., 1988; Degterev et al., 2005). Necroptosis is thought to be important in tumor regulation, including tumorigenesis, tumor immunity, and tumor metastasis (Stoll et al., 2017; Seehawer et al., 2018; Yan et al., 2021). Necroptosis plays a dual role in tumor regulation, not only promoting tumor progression and metastasis (Park et al., 2009; McCormick et al., 2016; Strilic et al., 2016) but also preventing tumor progression (Feng et al., 2015; Höckendorf et al., 2016). At present, the mechanism of necroptosis in tumor regulation is still unclear, and the research on the role of necroptosis-related lncRNAs in CC is not conclusive. This work aimed to use bioinformatics to investigate the role of necroptosis-related lncRNAs in CC and use qRT–PCR to verify the expression of some lncRNAs.

## Materials and Methods

### Data Collection

RNA sequencing (RNA-seq) data and clinical features were obtained from the TCGA database (https://portal.gdc.cancer.gov/repository) (Liu et al., 2018) on 22 September 2021, including 473 tumor datasets and 41 normal datasets. Data for 31 necroptosis-related genes were obtained from the GSEA website (https://www.gsea-msigdb.org/gsea) and prior reviews (Hanson, 2016; Chen et al., 2019; Molnár et al., 2019; Zhu et al., 2019; Liu et al., 2021b). Before comparison, the expression data were standardized to fragment per kilobase million (FPKM) values (Conesa et al., 2016).

### Selection of Necroptosis-Related lncRNAs and Expression Difference Analysis

The “limma” (Ritchie et al., 2015), “dplyr,” “ggalluvial,” and “ggplot2” packages were used to map the Sankey relational diagram for necroptosis genes and necroptosis-related lncRNAs. We used Pearson’s correlation analysis for filtering, and the criteria were |Pearson R| >0.4 and *p* < 0.001. The “survival” package (van Dijk et al., 2008) was used to select prognostic necroptosis-related lncRNAs with *p* < 0.05 using univariate Cox regression analysis, and a forest map was also drawn. The differential expression of necroptosis-related lncRNAs explored by univariate Cox regression analysis was expressed as a heat map and boxplot by the “limma,” “pheatmap,” “reshape2,” and “ggpubr” packages. DEGs (differentially expressed genes) are noted: * if *p* < 0.05, ** if *p* < 0.01, and *** if *p* < 0.001.

### Consensus Clustering and Immune Correlation Analysis for Prognostic Necroptosis-Related lncRNAs

Univariate Cox regression analysis was used to analyze the expression of necroptosis-related lncRNAs (Cristescu et al., 2015), and the “ConsensusClusterPlus” (Wilkerson and Hayes, 2010) and “limma” packages were used to divide all of the CC data into subgroups. The difference in survival probability was analyzed in all clusters. A heat map of the correlation between clusters and clinics was drawn. The expression and coexpression of immune checkpoint inhibitors (ICPis) (PD-1, PD-L1, CTLA-4), differences in the content of immune cells, and the immune scores (including the ESTMATE score, stromal score, and immune score) were also studied at the same time. DEGs (differentially expressed genes) are noted: * if *p* < 0.05, ** if *p* < 0.01, and *** if *p* < 0.001.

### Establishment of the Risk Model

The prognostic usefulness of prognostic necroptosis-related lncRNAs was investigated. Expression and clinical data in TCGA were used, and least absolute shrinkage and selection operator (LASSO) (Bunea et al., 2011) regression analysis {risk score = Ʃ [Exp (lncRNA) × coef (lncRNA)]} was used to create a predictive signature for necroptosis-related lncRNAs. The corresponding expression of the included lncRNAs is Exp (lncRNA), and the regression coefficient is coef (lncRNA). We randomized all samples at a 7:3 ratio into training and testing groups, and according to the median risk score, all samples were separated into two groups: high-risk and low-risk (Cox, 1972).

Survival analysis, receiver operating characteristic (ROC) curves (Hanley and McNeil, 1982), and the areas under the time-dependent ROC curves (AUC) were presented by “survival” and “survminer” packages. This study is useful in determining the model’s efficiency.

### Independent Prognostic Analysis and Principal Component Analysis

We analyzed whether clinical characteristics (age, sex, and TNM stage) can be used as independent prognostic factors. The “survival” package was used in a Cox regression-based univariate and multivariate independent prognostic study. According to the expression patterns of necroptosis-related lncRNAs, principal component analysis (PCA) was applied to weaken the dimensionality, identify the model, and visualize the high-dimensional data of the whole gene expression profile, necroptosis-related lncRNAs, necroptosis-related lncRNAs, and risk model. “limma” and “scatterplot3d” packages were used in this process.

### Functional Analysis

To identify the differentially expressed lncRNAs, we employed gene ontology (GO) analysis, which consisted of three parts: cellular component (CC), molecular function (MF), and biological process (BP). The “clusterProfiler” package was used in this process. The enrichment analysis of the Kyoto Encyclopedia of Genes and Genomes (KEGG) in the high- and low-risk groups was also investigated in this work (Damian and Gorfine, 2004). The “clusterProfiler,” “limma,” “org.Hs.e.g.db,” and “enrichplot” packages were applied in this process. *p* < 0.05 was considered to indicate significant functional enrichment.

### Nomogram and Survival Analysis of Different Clinical Features

A nomogram (Iasonos et al., 2008) was created to predict the survival of CC patients using the “rms” package. Expression and survival analyses of various clinicopathological characteristics were produced using the “limma” and “ggpubr” packages. This analysis is helpful to verify the validity of the model.

### Tumor Mutational Burden and Tumor Immune Analysis

To display the tumor mutational burden (TMB) of patients with CC, we utilized the “maftools” package to examine and integrate the TCGA data and analyzed the difference and survival of TMB between high- and low-risk groups. The “limma,” “ggpubr,” “survival,” “survminer,” and “maftools” packages were applied for this analysis. We investigated the relationship between the risk score and immune cells and predicted their correlation. The CIBERSORT algorithm was used in this study. The survival differences of high- and low-score patients in each immune cell type were analyzed by the “limma,” “survival,” and “survminer” packages. We studied the differential expression of immune checkpoints in the high- and low-risk groups. The variations in expression between the high- and low-risk groups and the disparities in survival between the high- and low-score groups with distinct immune pathways were investigated. The contrast between a high and low score was based on the median immune cell level or immune pathway enrichment level. The degree of difference was noted: * if *p* < 0.05, ** if *p* < 0.01, and *** if *p* < 0.001.

### Prediction of Potential Compounds in the Treatment of Colon Cancer

To predict the potential compounds that may be used in CC therapy, we calculated the IC50 values of the compounds obtained from the GDSC website. The compounds that may be used for CC therapy were predicted using the “pRRophetic,” “limma,” “ggpub,” and “ggplot2” packages.

### Quantitative Real-Time Polymerase Chain Reaction

The Shanghai Cell Bank of the Chinese Academy of Sciences provided the normal human colonic epithelial cell line NCM460 and the human CC cell line HT29. Six pairs of CC patients’ tumors and adjacent tissue samples were gathered from the First Affiliated Hospital of Nanchang University. Total RNA was obtained and purified using the TransZol Up Plus RNA Kit (TransGen, Beijing, PRC). EasyScript One-Step gDNA Removal and cDNA Synthesis SuperMix (TransGen, Beijing, PRC) and T100 Thermal Cycler (BIO-RAD, United States) were used for reverse transcription. PerfectStart Green qPCR SuperMix (TransGen, Beijing, PRC) and CFX Connect Optics Module (BIO-RAD, United States) were used for qRT–PCR. All experimental operations followed the steps of the product manual. The primer sequences for PCR amplification were as follows:

FALEC, forward: 5′-CCT​GGC​CAA​GAA​GCT​CAT​AC-3′,

reverse: 5′-TGA​GGA​CAC​CGA​CTA​CTG​AGA​A-3′;

ZEB1-AS1, forward: 5′-GGT​TTC​CTT​CCT​GCT​TCC​CA-3′,

reverse: 5′-ACT​CCG​GTC​ACG​TTT​CAG​TT-3′;

MYOSLID, forward: 5′-TGG​TGG​GAT​CTG​GAA​GAA​GC-3′,

reverse: 5′-TCA​GCC​ATG​TCC​TTG​CCT​TC-3′;

MACORIS, forward: 5′-CTC​CAA​GAG​GGA​AGG​AGG​GA-3′,

reverse: 5′-AGC​TCA​CTC​CCA​GTG​GGT​AA-3′;

ZKSCAN2-DT, forward: 5′-TCT​GGC​GGA​AGT​ATC​TGT​GC-3′,

reverse: 5′-AGC​ACC​AGA​AGA​GAG​CAA​GC-3′;

LCMT1-AS1, forward: 5′-GGC​AGA​GAA​TCC​AGC​CAG​AA-3′,

reverse: 5′-TTC​TGG​GAC​CAG​CAG​GTA​GA-3′;

GAPDH, forward: 5′-GGG​AAG​GTG​AAG​GTC​GGA​GT-3′,

reverse: 5′-GGG​GTC​ATT​GAT​GGC​AAC​A-3′.

Each sample was replicated three times, and GAPDH was used as the internal control. The 2^^−ΔΔCt^ method was used to determine the relative expression levels. The differences in FALEC, ZEB1-AS1, MYOSLID, MACORIS, ZKSCAN2-DT, and LCMT1-AS1 expression were tested by *t*-tests. GraphPad Prism (version 8.0.2) was used to create the graphs (* if *p* < 0.05, ** if *p* < 0.01, and *** if *p* < 0.001).

### Statistical Analysis

For statistical analysis and outcome display, R software (version 4.1.0) was utilized. The Benjamini–Hochberg method was utilized to authenticate the differential expressions. The Mann–Whitney *U* test was utilized to detect the mRNA level of pyroptosis-related lncRNAs. Student’s *t*-test was utilized to determine the differences between the two groupings. The classification variables in the training and testing tests were contrasted using the chi-square test. The link between subtypes, clinicopathological factors, risk score, immune check inhibitors, and immune infiltration levels was assessed using the Pearson correlation test. The Kaplan–Meier method with a two-sided log-rank test was employed for survival analysis.

## Result

### Prognosis-Related lncRNAs With Coexpression of Necroptosis

**FIGURE 1:**
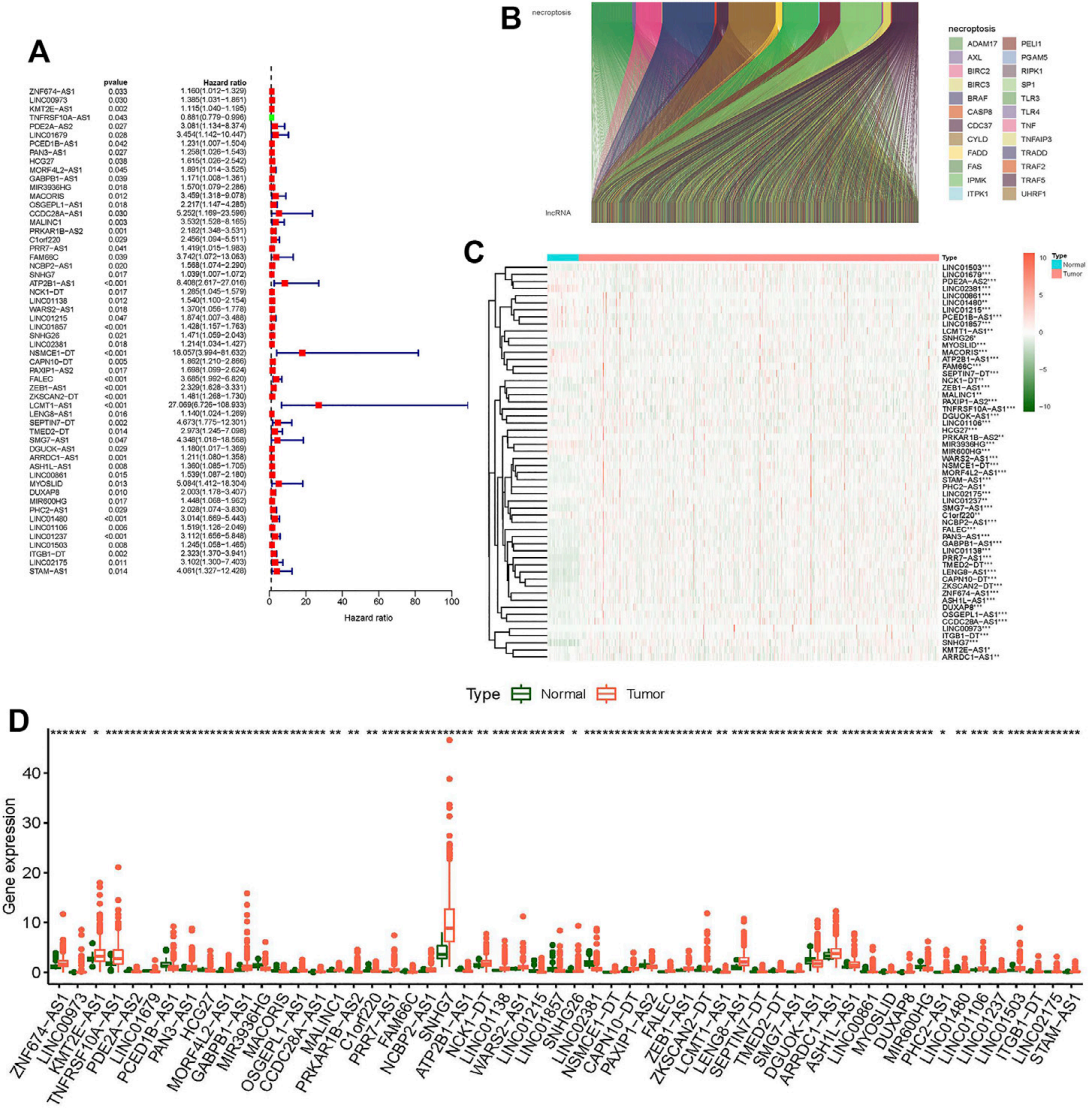
Differentially expressed genes of necroptosis-related lncRNAs in colon tumor and normal tissues. **(A)** Forest plot shows prognostic-related genes of necroptosis-related lncRNAs. **(B)** Sankey relational diagram for necroptosis genes and necroptosis-related lncRNAs. **(C)** Heat map shows differentially expressed genes of necroptosis-related lncRNAs. **(D)** Boxplot shows differentially expressed genes of necroptosis-related lncRNAs. *p < 0.05, **p < 0.01, ***p < 0.001.

We confirmed 556 lncRNAs with a coexpression relationship in CC (|Pearson R| > 0.4 and *p* < 0.001) (Figure 1B). Univariate Cox analysis (*p* < 0.05) was used to select 56 differentially expression prognosis associated lncRNAs: ZNF674-AS1, LINC00973, KMT2E-AS1, TNFRSF10A-AS1, PDE2A-AS2, LINC01679, PCED1B-AS1, PAN3-AS1, HCG27, MORF4L2-AS1, GABPB1-AS1, MIR3936HG, MACORIS, OSGEPL1-AS1, CCDC28A-AS1, MALINC1, PRKAR1B-AS2, C1orf220, PRR7-AS1, FAM66C, NCBP2-AS1, SNHG7, ATP2B1-AS1, NCK1-DT, LINC01138, WARS2-AS1, LINC01215, LINC01857, SNHG26, LINC02381, NSMCE1-DT, CAPN10-DT, PAXIP1-AS2, FALEC, ZEB1-AS1, ZKSCAN2-DT, LCMT1-AS1, LENG8-AS1, SEPTIN7-DT, TMED2-DT, SMG7-AS1, DGUOK-AS1, ARRDC1-AS1, ASH1L-AS1, LINC00861, MYOSLID, DUXAP8, MIR600HG, PHC2-AS1, LINC01480, LINC01106, LINC01237, LINC01503, ITGB1-DT, LINC02175, and STAM-AS1 (Figures 1A,C,D).

### Consensus Cluster Analysis of Prognosis Necroptosis-Related lncRNAs

**FIGURE 2:**
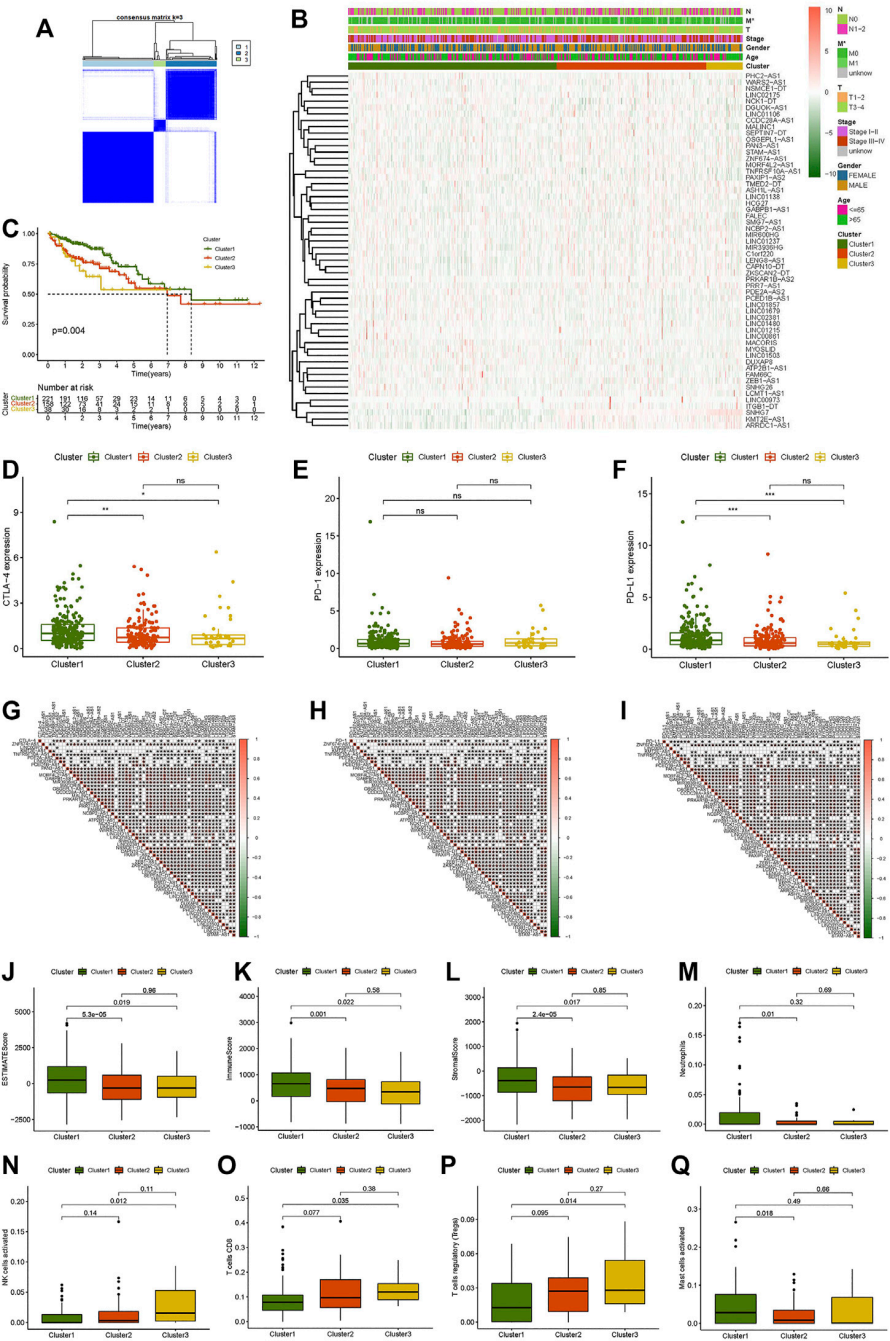
Consensus clustering analysis of necroptosis-related lncRNAs and immune correlation analysis. **(A)** Consensus clustering matrix for k = 3. **(B)** Heat map and clinicopathologic features of Clusters 1, 2, and 3. **(C)** Kaplan–Meier curves of overall survival (OS) of Clusters 1, 2, and 3. **(D–F)** CTLA-4, PD-1, and PD-L1 expression levels of Clusters 1, 2, and 3. **(G–I)** Correlation between CTLA-4, PD-1, and PD-L1 expression levels and differential expression of necroptosis-related lncRNAs. **(J)** ESTMATE score. **(K)** Immune score. **(L)** Stromal score. **(M)** Neutrophils. **(N)** Activated NK cells. **(O)** CD8 T cells. **(P)** Regulatory T cells (Tregs). **(Q)** Activated mast cells. *p < 0.05, **p < 0.01, ***p < 0.001.

To investigate the connections between the expression levels of 56 prognostic necroptosis-related lncRNAs and CC subtypes, we performed a consensus clustering analysis with all 473 CC samples in the TCGA cohort. The tumor samples were divided into clusters via the “ConsensusClusterPlus” package. By increasing the clustering variable (k) from 2 to 9, we found that when k = 3, the intragroup correlations were the highest, and the intergroup correlations were the lowest (Supplementary Figures S2A–C, Figure 2A). Survival analysis indicated remarkable differences in survival among the three subgroups, and Cluster 1 had a better survival probability than Cluster 2 and Cluster 3 (Figure 2C). The gene expression and clinical characteristic features are presented in a heat map (Figure 2B), which showed that the difference in metastasis (M) was statistically significant among the three clusters. The expression differences of immune checkpoints (PD-1, PD-L1, CTLA-4) in the three clusters and the expression differences between the tumor and normal tissues were explored. The outcome showed that the expression levels of CTLA-4 and PD-L1 were higher in Cluster 1 than Clusters 2 and 3 (Figures 2D–F), and the CTLA-4 expression was substantially greater in tumor tissues than in normal tissues (Supplementary Figures S3W–Y). The difference analysis of the infiltration fractions of 22 immune cells (B cells memory, B cells naive, dendritic cells activated, dendritic cells resting, eosinophils, macrophages M0, macrophages M1, macrophages M2, mast cells activated, mast cells resting, neutrophils, monocytes, NK cells activated, NK cells resting, plasma cells, T cells CD4 memory activated, T cells CD4 memory resting, T cells CD4 naive, T cells CD8, T cells follicular helper, T cells gamma delta, and T cells regulatory (Tregs)) (Supplementary Figures S3A–V) showed the following: neutrophils, NK cells activated, T cells CD8, T cells regulatory, and mast cells activated had obvious difference between subgroups (Figures 2M–Q). The differences in the ESTMATE score, stromal score, and immune score between Cluster 1 and the other clusters were statistically significant (Figures 2J–L). Cluster 1 had a higher immunological infiltration level than Clusters 2 and 3. The correlation of expression level between the 56 necroptosis-related lncRNAs and immune checkpoints showed the following: the expression level of CTLA-4 was related to TNFRSF10A-AS1, PDE2A-AS2, LINC01679, PCED1B-AS1, HCG27, GABPB1-AS1, MACORIS, PRKAR1B-AS2, C1orf220, PRR7-AS1, FAM66C, SNHG7, ATP2B1-AS1, LINC01138, LINC01215, LINC01857, SNHG26, LINC02381, PAXIP1-AS2, ZEB1-AS1, LCMT1-AS1, TMED2-DT, SMG7-AS1, LINC00861, MYOSLID, MIR600HG, PHC2-AS1, LINC01480, LINC01237, and LINC01503 (Figure 2G); the expression level of PD-1 was related to PDE2A-AS2, LINC01679, PCED1B-AS1, PAN3-AS1, HCG27, MORF4L2-AS1, MIR3936HG, MACORIS, OSGEPL1-AS1, MSALINC1, NCBP2-AS1, WARS2-AS1, LINC01215, LINC01857, LINC02381, PAXIP1-AS2, FALEC, ZKSCAN2-DT, LCMT1-AS1, LENG8-AS1, DGUOK-AS1, ASH1L-AS1, LINC00861, PHC2-AS1, LINC01480, LINC01106, LINC01503, and STAM-AS1 (Figure 2H); and the expression level of PD-L1 was related to LINC00973, KMT2E-AS1, TNFRSF10A-AS1, PDE2A-AS2, LINC01679, PCED1B-AS1, HCG27, GABPB1-AS1, MACORIS, PRKAR1B-AS2, PRR7-AS1, FAM66C, SNHG7, ATP2B1-AS1, LINC01138, LINC01215, LINC01857, SNHG26, LINC02381, NSMCE1-DT, PAXIP1-AS2, ZEB1-AS1, LCMT1-AS1, LENG8-AS1, TMED2-DT, ARRDC1-AS1, LINC00861, MYOSLID, PHC2-AS1, LINC01480, LINC01503, and LTGB1-DT (Figure 2I). Necroptosis-related lncRNAs showed a strong correlation with tumor immunity in CC, suggesting that necroptosis-related lncRNAs may provide a new reference marker for immunotherapy of CC.

### Construction and Validation of a Risk Model According to Necroptosis-Related lncRNAs in Colon Cancer

The 473 tumor sample data points from TCGA were randomly split into two groups at a 7:3 ratio. We constructed a LASSO regression model based on univariate Cox regression analysis to predict the survival of CC (Supplementary Figures S2D,E). Six lncRNAs (MACORIS, FALEC, ZEB1-AS1, ZKSCAN2-DT, LCMT1-AS1, and MYOSLID) were identified for further analysis. The risk score was calculated as follows: risk score = (0.142490623 * MACORIS exp.) + (0.279155631 * FALEC exp.) + (0.195713675 * ZEB1-AS1 exp.) + (0.031055692 * ZKSCAN2-DT exp.) + (0.452830425 * LCMT1-AS1 exp.) + (0.446685498 * MYOSLID exp.). Each sample’s risk score was separated into high-risk and low-risk groups based on the median risk score in the training and testing groups.

**FIGURE 3:**
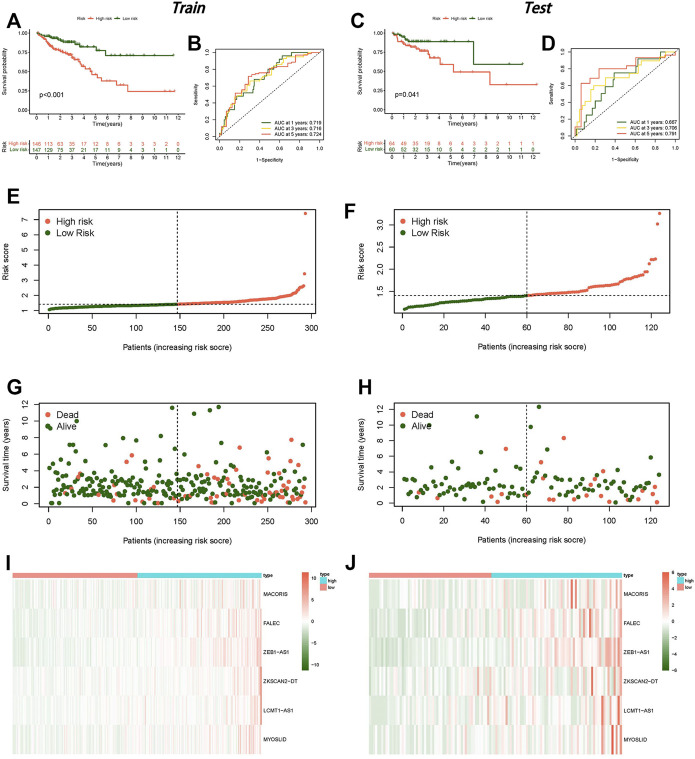
Establishment of a risk model for necroptosis-related lncRNAs in colon cancer. **(A)** Kaplan–Meier curve for OS in the training group. **(B)** ROC curve in the training group. **(C)** Kaplan–Meier curve for OS in the testing group. **(D)** ROC curve in the testing group. **(E)** Risk score distribution in the training group. **(F)** Risk score distribution in the testing group. **(G)** OS status in the training group. **(H)** OS status in the testing group, **(I)** Heat map in the training group. **(J)** Heat map in the testing group.

The low-risk group had a better prognosis than the high-risk group according to the survival analysis, and the difference was statistically significant (Figures 3A,C). The AUCs of the training and testing cohorts were 0.719 and 0.667 at 1 year, 0.716 and 0.706 at 3 years, and 0.724 and 0.791 at 5 years (Figures 3B,D), implying that the risk scores performed well in terms of prediction. Clearly, the risk curve revealed that the lower the risk score was, the lower the patient survival rate (Figures 3E–H). The heat map revealed that the expression of the six lncRNAs was higher in the high-risk group than in the low-risk group (Figures 3I,J).

**FIGURE 4:**
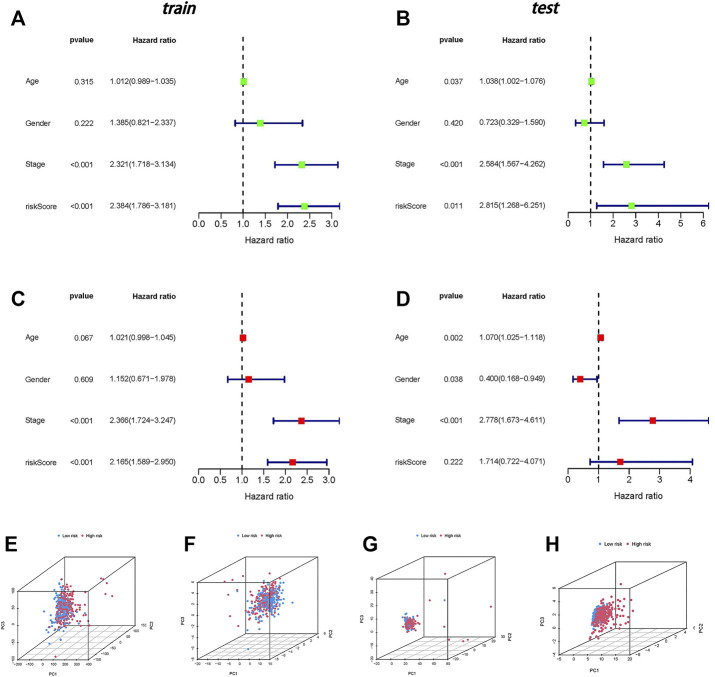
Independent prognostic analysis and PCA. **(A)** Univariate independent prognostic analysis in the training group. **(B)** Univariate independent prognostic analysis in the testing group. **(C)** Multivariate independent prognostic analysis in the training group. **(D)** Multivariate independent prognostic analysis in the testing group. **(E)** PCA of all genes. **(F)** PCA of necroptosis genes. **(G)** PCA of necroptosis-related lncRNAs. **(H)** PCA of risk lncRNAs.

The results of univariate and multivariate Cox analyses are shown in a forest plot (Figures 4A–D). The stage and risk score were independent predictors of poor survival in both groups, according to the univariate Cox regression analysis, and the stage was a predictive factor for individuals with CC in both groups, according to the multivariate analysis.

### Principal Component Analysis Validates the Grouping Ability of the Risk Model

In four expression profiles (entire gene expression profiles, necroptosis genes, necroptosis-related lncRNAs, and the risk model classified by the expression profiles of the six necroptosis-related lncRNAs), we employed PCA to examine the differences between the low- and high-risk groups (Figures 4E–H). The results showed that six necroptosis-related lncRNAs had the best discrimination ability and could distinguish between the low- and high-risk groups quite well.

### Gene Ontology and Kyoto Encyclopedia of Genes and Genomes Analysis

**FIGURE 5:**
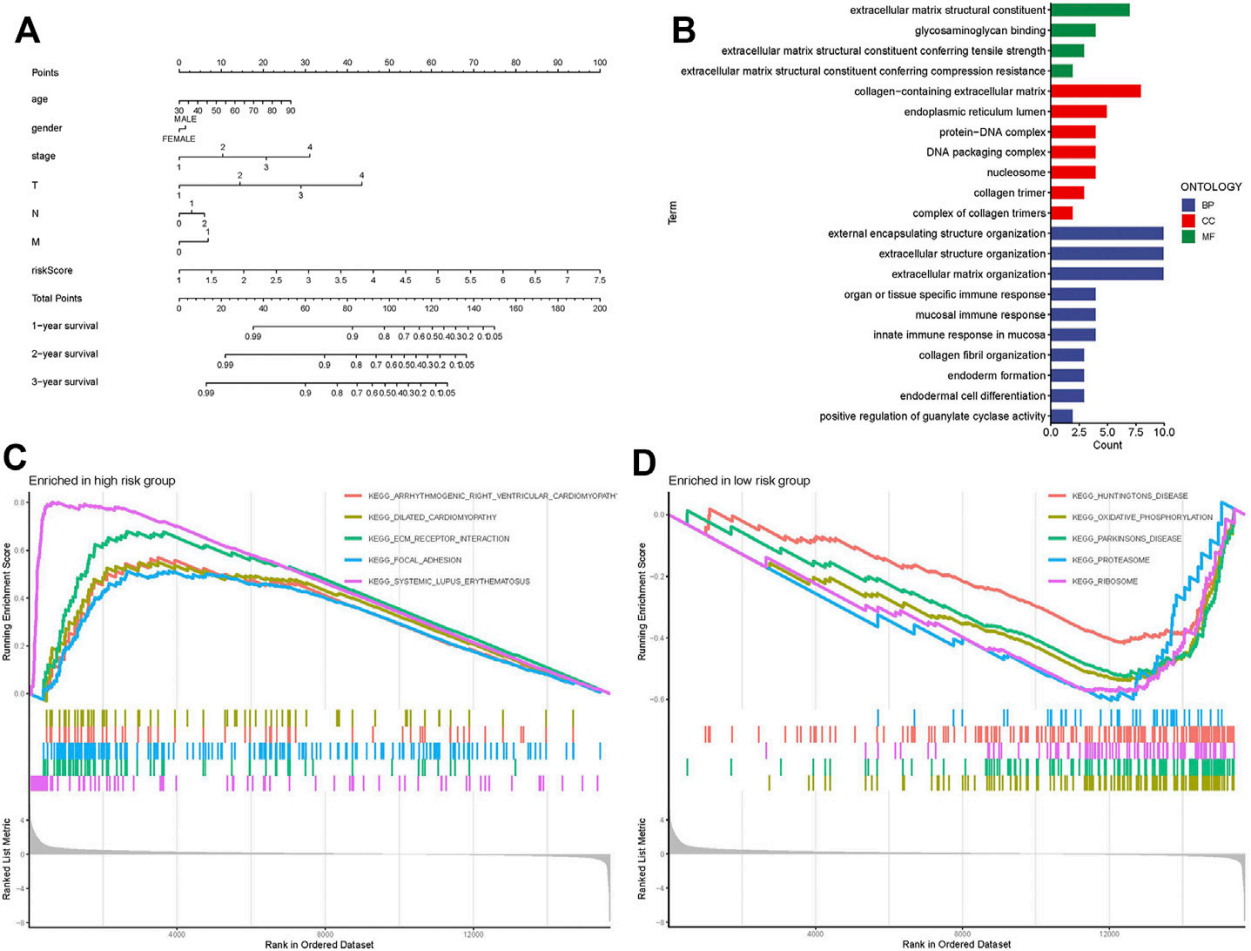
Nomogram, GO, and KEGG analysis. **(A)** Nomogram. **(B)** GO enrichment analysis in risk genes. **(C)** KEGG enrichment analysis in the high-risk group. **(D)** KEGG enrichment analysis in the low-risk group.

GO analysis indicated that necroptosis-related lncRNAs were closely correlated with intracellular and extracellular structural stability and cellular metabolism (Figure 5B). The KEGG enrichment analysis results are also displayed (Figures 5C,D): some cancer- and metabolism-related pathways were enriched by gene set enrichment analyses, including ECM receptor interaction and focal adhesion pathway that were significantly associated with the high-risk group. Oxidative phosphorylation, proteasome, and ribosome pathway were significantly associated with the low-risk group. A nomogram was established to predict the survival rate of CC patients (Figure 5A).

### Group Verification of the Model and the Clinicopathological Correlation

**FIGURE 6:**
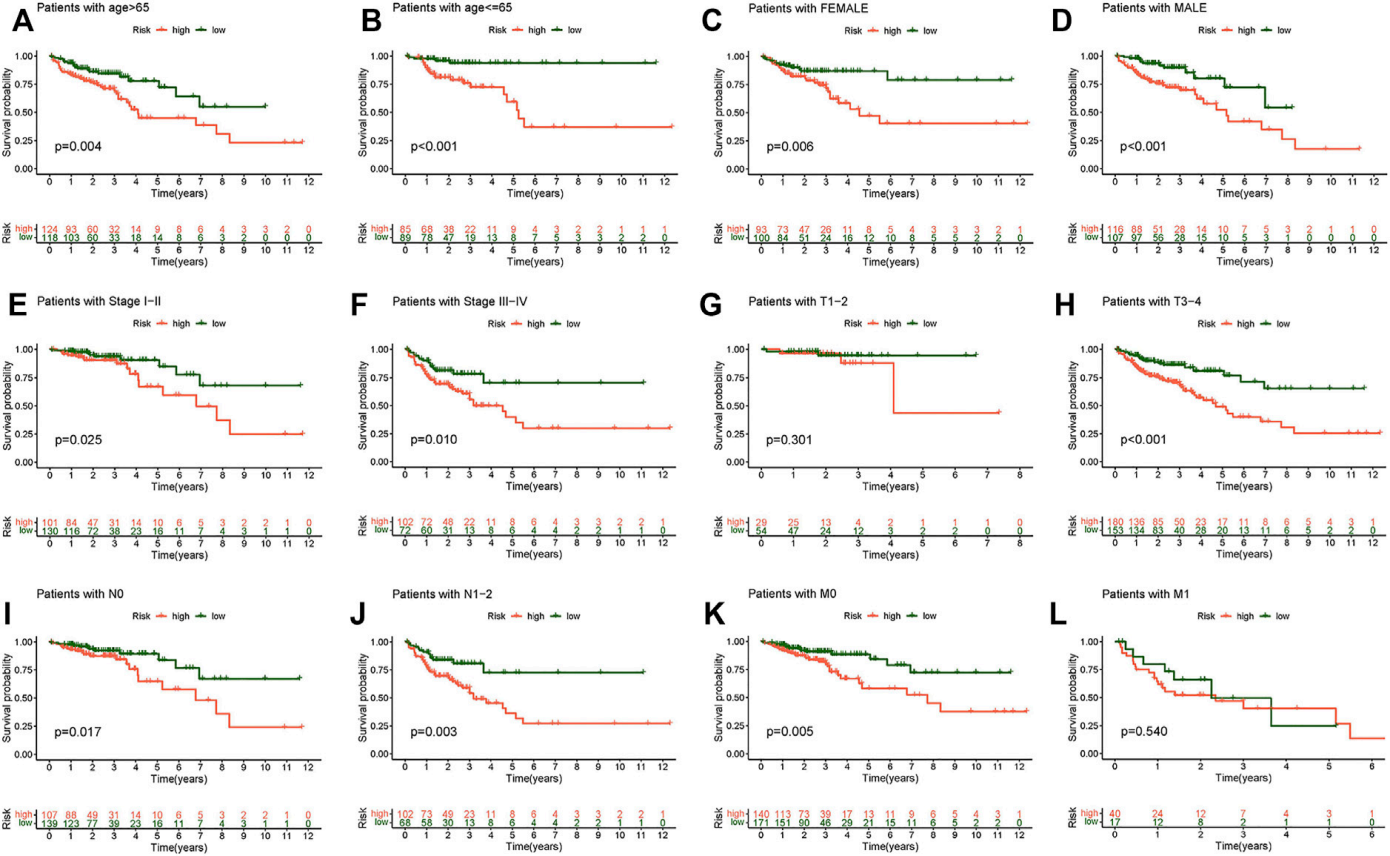
Kaplan–Meier survival subgroup analysis for differential clinicopathological features in the high- and low-risk score group. **(A)** Patients with age >65. **(B)** Patients with age ≤65. **(C)** Female patients. **(D)** Male patients. **(E)** Patients with stages I-II. **(F)** Patients with stages III-IV. **(G)** Patients with stages T1-2. **(H)** Patients with stages T3-4. **(I)** Patients with stage N0. **(J)** Patients with stages N1-2. **(K)** Patients with stage M0. **(L)** Patients with stage M1.

**FIGURE 7:**
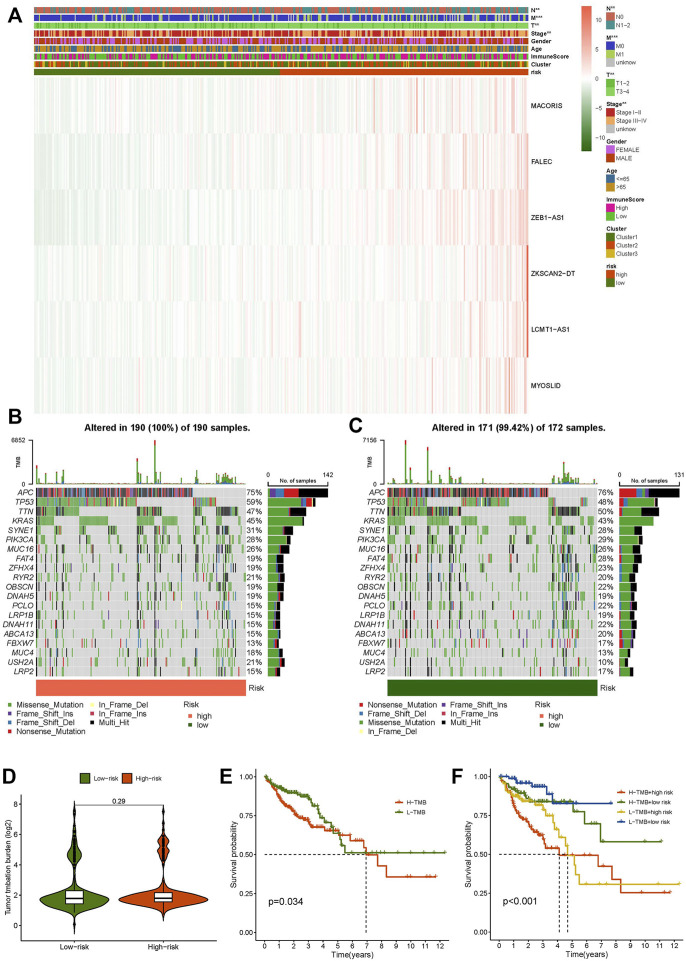
Clinicopathological features in the high- and low-risk score group, TME analysis. **(A)** Heat map of clinicopathological features in the high- and low-risk group. **(B)** Waterfall plot displays mutation information of the genes with high mutation frequencies in the high-risk group. **(C)** Waterfall plot displays mutation information of the genes with high mutation frequencies in the low-risk group. **(D)** TMB difference in the high- and low-risk patients. **(E)** Kaplan–Meier curve analysis of OS is shown for patients in high and low TMB. **(F)** Kaplan–Meier curve analysis of OS is shown for patients classified according to the TMB and necroptosis-related lncRNA model.

The relationship between the survival probability and the risk score in different clinicopathological characteristics was also investigated. The results revealed that patients in the low-risk group had a much higher survival rate overall (Figure 6). This result confirms the reliability of the model. A heat map showed the relationship between the differential expression of six necroptosis-related lncRNAs and clinicopathological features. These results showed that high-risk patients were significantly correlated with stage, T stage, N stage, and M stage (Figure 7A), and there was no effective difference in the expression of immune checkpoints between the low- and high-risk groups. This model can be used to help guide the diagnosis, prognostication, and treatment strategies (* if *p* < 0.05, ** if *p* < 0.01, and *** if *p* < 0.001).

### Tumor Mutational Burden and Immune Correlation Analysis

We discovered that the TMB of the high-risk group was higher than that of the low-risk group based on the TMB score generated from the somatic mutation data of TGCA (Figures 7B,C). However, overall, the TMB did not differ significantly between the two groups (Figure 7D). The samples were divided into high- and low-mutation groups according to the TMB score. The low-mutation group had a higher survival rate than the high-mutation group, according to the survival analysis (Figures 7E,F). We further tested whether the necroptosis-related lncRNA model can better predict survival than TMB, and the results showed that the model’s prediction ability was unquestionably higher than that of TMB (Figure 7F).

**FIGURE 8:**
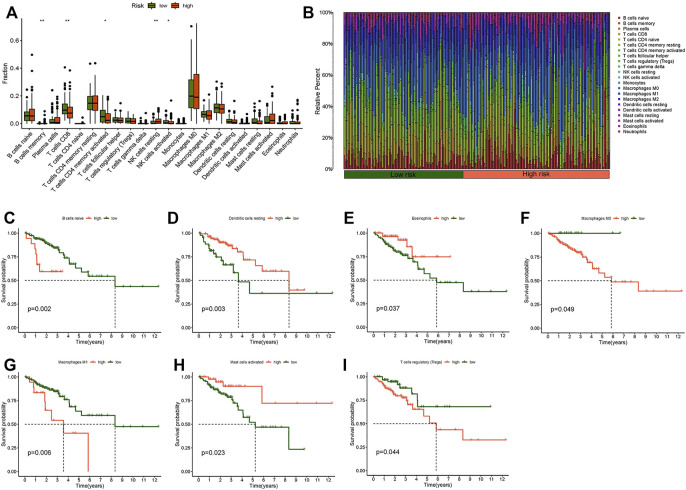
Immune cell infiltration analysis. **(A)** Boxplot of difference analysis of immune cells in the high- and low-risk group. **(B)** Relative percent of different immune cells in the high- and low-risk group. **(C)** Kaplan–Meier survival analysis in naive B cells. **(D)** Kaplan–Meier survival analysis in resting dendritic cells. **(E)** Kaplan-Meier survival analysis in eosinophils. **(F)** Kaplan–Meier survival analysis in M0 macrophages. **(G)** Kaplan–Meier survival analysis in M1 macrophages. **(H)** Kaplan–Meier survival analysis in activated mast cells. **(I)** Kaplan–Meier survival analysis in regulatory T cells (Tregs). *p < 0.05, **p < 0.01, ***p < 0.001.

We used the “limma,” “reshape2,” and “ggpubr” packages for immune cell infiltration analysis. The filtering standard was *p* < 0.05. B cells memory, activated NK cells, resting NK cells, naive T cells CD4, and CD8 T cells had obvious correlations with the risk score (Figure 8A). A bar plot was used to show the percentage of each immune cell in the high-risk and low-risk groups (Figure 8B). We also researched the survival of immune cells: naive B cells, resting dendritic cells, eosinophils, M0 macrophages, M1 macrophages, activated mast cells, and regulatory T cells (Tregs) showed significant differences between the low- and high-risk groups in survival probability (Figures 8C–I). The prognosis of the low-score group was significantly better than that of the high-score group in B cells naive, macrophages M0, macrophages M1, and T cells regulatory (Tregs). The survival probability of the high score group was better than the low-score group in dendritic cells resting, eosinophils, and mast cells activated.

**FIGURE 9:**
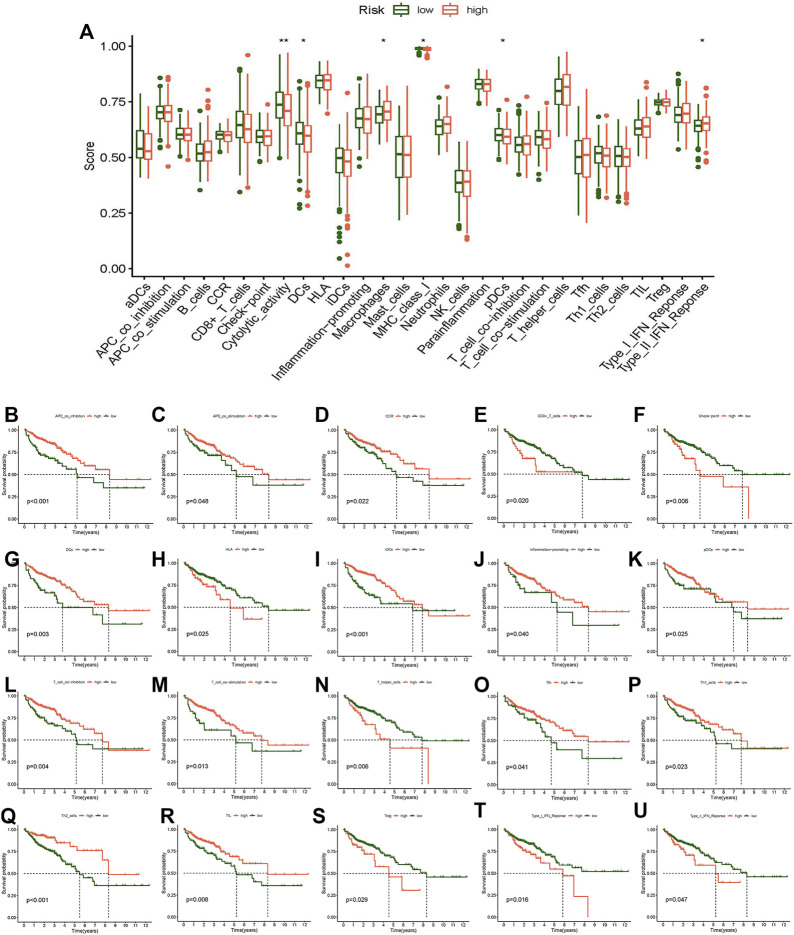
Analysis of the difference in immune-related function and survival analysis. **(A)** Boxplot of the difference of immune-related function. **(B)** Kaplan–Meier survival analysis in APC co-inhibition. **(C)** Kaplan–Meier survival analysis in APC co-stimulation. **(D)** Kaplan–Meier survival analysis in CCR. **(E)** Kaplan–Meier survival analysis in CD8^+^ T cells. **(F)** Kaplan–Meier survival analysis in checkpoint. **(G)** Kaplan–Meier survival analysis in DCs. **(H)** Kaplan–Meier survival analysis in HLA. **(I)** Kaplan–Meier survival analysis in iDCs. **(J)** Kaplan-Meier survival analysis in inflammation-promoting. **(K)** Kaplan–Meier survival analysis in pDCs. **(L)** Kaplan–Meier survival analysis in T cell co-inhibition. **(M)** Kaplan–Meier survival analysis in T cell co-stimulation. **(N)** Kaplan–Meier survival analysis in T helper cells. **(O)** Kaplan–Meier survival analysis in Tfh. **(P)** Kaplan–Meier survival analysis in Th1 cells. **(Q)** Kaplan–Meier survival analysis in Th2 cells. **(R)** Kaplan–Meier survival analysis in TIL. **(S)** Kaplan–Meier survival analysis in Tregs. **(T)** Kaplan–Meier survival analysis in Type I IFN response. **(U)** Kaplan–Meier survival analysis in Type II IFN response. *p < 0.05, **p < 0.01, ***p < 0.001.

In addition, we researched the relationship between the risk score and immune pathway in CC by the “limma,” “GSVA,” “GSEABase,” “ggpubr,” and “reshape2” packages. Boxplots of the results showed that cytolytic activity, DCs, macrophages, MHC class I, pDCs, and type II IFN response were significantly different in the risk score (Figure 9A). Survival analysis between different immune functions showed (Figure 9B–U) that the low-score group has better survival than the high-score group in CD8^+^ T cells, checkpoint, HLA, T helper cells, Tregs, type I IFN response, and type II IFN response; the high-score group has better survival than the low-score group in APC co-inhibition, APC co-stimulation, CCR, DCs, iDCs, inflammation-promoting, pDCs, T cell co-inhibition, T cell co-stimulation, Tfh, Th1-cells, Th2-cells, and TIL. These findings indicate that the risk score for various immune functions has a considerable impact on CC risk. These results may be helpful to guide individualized immunotherapy.

### Prediction of Potential Chemical Drugs for the Treatment of Cellular Component

**FIGURE 10:**
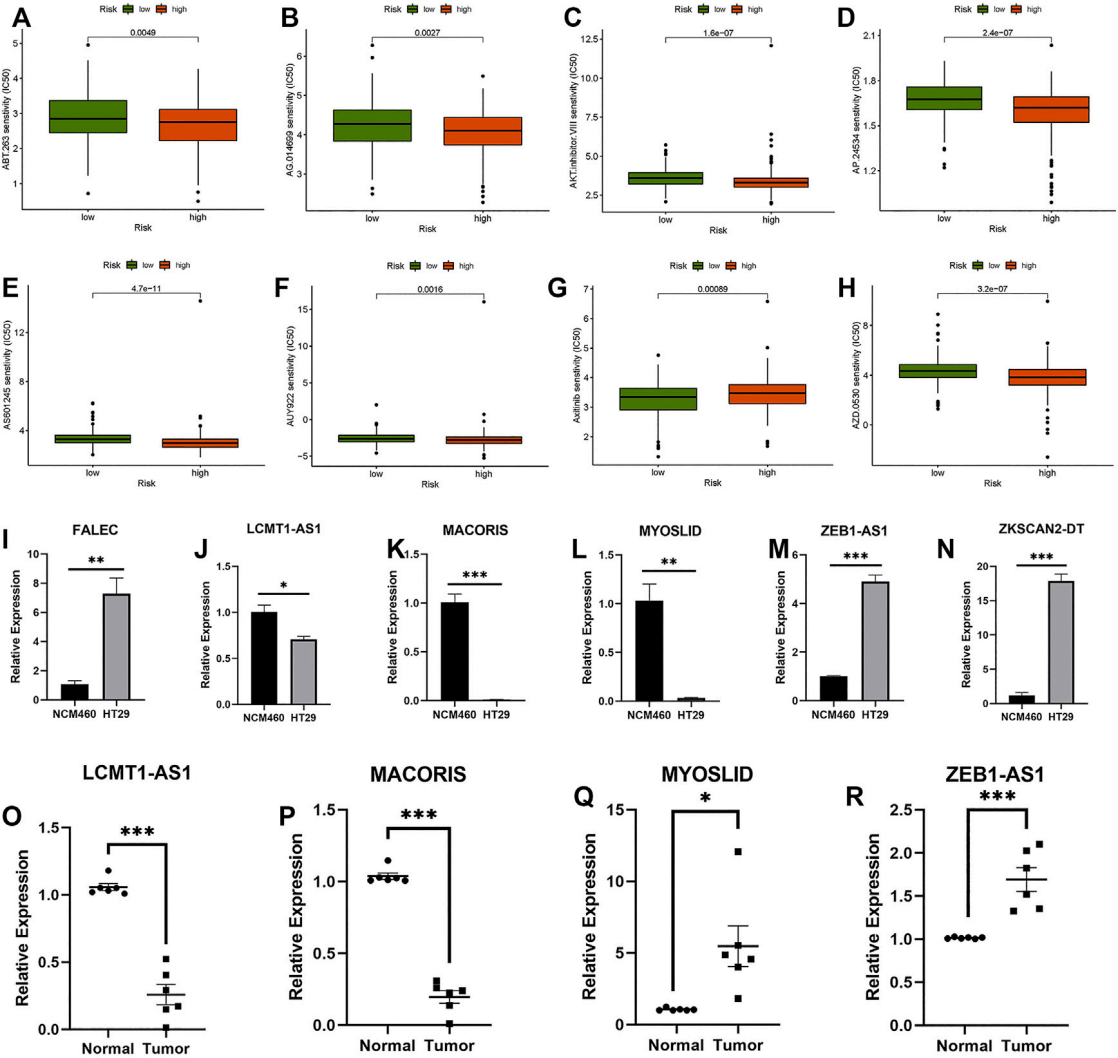
Prediction of potential compounds in the treatment of CC and qRT–PCR. **(A)** ABT.263. **(B)** AG.014699. **(C)** AKT.inhibitor.VII senstivity. **(D)** AP.24534. **(E)** AS601245. **(F)** AUY922. **(G)** Axitinib. **(H)** AZD.0530. **(I–N)** Relative expression of FALEC, LCMT1-AS1, MACORIS, MYOSLID, ZEB1-AS1, and ZKSCAN2-DT in NCM460, and HT29 cell line. **(O–R)** Relative expression of LCMT1-AS1, MACORIS, MYOSLID, and ZEB1-AS1 in normal and tumor tissue. *p < 0.05, **p < 0.01, ***p < 0.001.

The prediction of potential therapeutic drugs showed that the sensitivity to eight chemicals in the low- and high-risk groups differed significantly. Seven compounds (ABT.263, AG.014699, AKT.inhibitor, AP.24534, AS601245, AUY922, and AZD.0530) in the high-risk group had a higher sensitivity than those in the low-risk group (Figures 10A–F,H), and one compound (axitinib) had a higher sensitivity in the low-risk group than in the high-risk group (Figure 10G). These findings may be helpful for clinical treatment.

### Analysis of Quantitative Real-Time Polymerase Chain Reaction

Six necroptosis-related lncRNAs (FALEC, LCMT1-AS1, MACORIS, MYOSLID, ZEB1-AS1, and ZKSCAN2-DT) were selected. These lncRNAs were tested in NCM460 and HT29 cells. Because the expression level of FALEC and ZKSCAN2-DT in the tissues we obtained was too low, the credibility of the results of qRT–PCR was low. Only four lncRNAs (LCMT1-AS1, MACORIS, MYOSLID, and ZEB1-AS1) were tested in the CC adjacent tissues and tumor tissues using qRT–PCR. As shown in Figures 10I–R, the expression levels of these lncRNAs differed significantly between tumor and normal cells. This means that these factors are differentially expressed between tumor and normal colon cells. However, for lncRNA MYOSLID (Figures 10L–Q), the results in tissues and cell lines were different. Considering that the expression level in the cell line does not fully represent the TCGA sequencing data, the qRT–PCR results in the tissue are more reliable. On the whole, the experimental results also confirmed the reliability of the risk model. (* if *p* < 0.05, ** if *p* < 0.01, and *** if *p* < 0.001).

## Discussion

Colon cancer (CC) is one of the most common malignancies around the world. Its prevalence among young people has risen steadily in recent years (Islami et al., 2021). Many lncRNAs play a regulatory role in the onset and progression of CC (Liu et al., 2021a). Professor Chen confirmed that, in colon cells, lncRNA CCAT2 induces chromosome instability through BOP1-AURKB signals, thus promoting the occurrence of cancer (Chen et al., 2020). Kawasaki found that lncRNA CALIC promotes metastasis of CC by upregulating AXL (Kawasaki et al., 2019). Wang confirmed that lncRNA LOC441461 promotes tumor growth and mobility in colorectal cancer cells through the RhoA/ROCK signaling pathway (Wang et al., 2020). An increasing number of studies have emphasized the crucial role of lncRNAs in CC in recent years, but the relationship between them is not entirely clear, and it is a hot topic of research.

Necroptosis is a type of programmed death. Its morphological characteristics are similar to those of necrosis, such as cell swelling, organelle dysfunction, and plasma membrane rupture (Weinlich et al., 2017; Seo et al., 2021). Currently, necroptosis has been demonstrated to play a significant role in tumor progression, and it is also associated with tumor immunity (Seifert et al., 2016; Snyder et al., 2019). McComb confirmed that necroptosis-associated molecules (CIAPs) play an essential role in reducing macrophage planned necrosis, which aids in pathogen management (McComb et al., 2012). The role of RIP3 (necroptosis-related molecules) in increasing myeloid cell-induced adaptive immune suppression and improving the proliferation of premalignant intestinal epithelial cells (IECs) was demonstrated by Liu’s research (Liu et al., 2019). Hoecke used a mouse model to prove that the necroptosis mediator MLKL has an antitumor immunity effect (Van Hoecke et al., 2020). Several studies have suggested that necroptosis plays an essential role in tumors, but the mechanism is still not fully defined. In our study, necroptosis-related lncRNAs were divided into different subgroups to construct prognostic markers for the first time and to systematically study the correlation between the tumor microenvironment, immune cell infiltration, immune checkpoints, and pyroptosis-related lncRNAs. We look forward to using these results to guide clinical diagnosis and treatment in the future.

Our research identified 31 genes related to necroptosis from the previous literature and the GEEA website. RNA expression files and clinical files were downloaded from the TCGA database. We first carried out coexpression analysis to identify the necroptosis-related lncRNAs, and 56 prognosis-related lncRNAs were ruled out using univariate Cox analysis. Consensus clustering analysis divided these lncRNAs into three subgroups. Survival analysis showed that Cluster 1 had better survival than the other groups (*p* = 0.004). For the expression of immune checkpoints, Cluster 1 was substantially higher than that of Clusters 2 and 3 for CTLA-4 and PD-L1. The expression of these three immunological checkpoints had a significant relationship with prognosis-related lncRNAs, which was the same as the immune score. These findings implied that consensus clustering is closely related to the prognosis of patients and may be related to the immune microenvironment. This might offer fresh insights into immunotherapy for CC patients. The prognostic model was constructed by LASSO regression, and six prognosis-related lncRNAs (MACORIS, FALEC, ZEB1-AS1, ZKSCAN2-DT, LCMT1-AS1, and MYOSLID) were used to establish the risk model. ROC curves indicated that the necroptosis-related lncRNA prognostic signature was highly accurate and reliable, and the AUC value in the training group was 0.719 at 1 year. Compared with other published lncRNA risk models in colon cancer, Zhang’s risk model AUC value was 0.662 at 1 year (Zhang et al., 2021) and Xing’s risk model AUC value was 0.6128 at 1 year (Xing et al., 2021). Our lncRNA risk model is more reliable than other published models. Clinicopathological analysis, survival analysis, PCA, TMB, and qRT–PCR analysis implied that this model offers high sensitivity for survival prediction. In addition, the analysis revealed that the signature might be employed as an independent factor in prognostication. Through analysis of the markers and the level of tumor immunity and immune cells, it was proven that these markers play an important role in CC. These findings point to the possibility that necroptosis-related lncRNAs are related to tumor immune infiltration. Therefore, the survival analysis of immune cells and the analysis of immune-related functions (including difference analysis and survival analysis) were also included in our study. The results of the survival analysis showed significant differences. The prediction of potential disease compounds may be helpful for clinical treatment. These studies may provide a meaningful reference for new targets of immunotherapy in the future.

In previous studies, Zheng’s research confirmed that FALEC is significantly expressed in endometrial carcinoma tissues and cells and plays a role in cell proliferation and cell cycle regulation (Zheng et al., 2019). Han’s study found that MYOSLID plays a key role in the incidence and progression of gastric cancer through the MYOSLID-miR-29c-3p-MCL-1 axis (Han et al., 2019). By sponging miR-455-3p, ZEB1-AS1 enhances PAK2 expression, enabling CC cell proliferation and metastasis (Ni et al., 2020). Necroptosis-related lncRNAs have been implicated in the occurrence and progression of malignancies in an increasing number of studies, but in CC, there is still insufficient research in this area. In this study, we firstly performed consensus clustering analysis of lncRNAs associated with necroptosis in CC and established a risk model for the first time. Second, our research systematically analyzed the relationship between necroptosis-related lncRNA prognostic markers and TMB, the tumor microenvironment, and immune cell infiltration for the first time, which may provide new ideas for further study of the predictive role of necroptosis-related lncRNAs markers in immunotherapy. Third, we predicted some potential compounds that may be used in the treatment of CC, which may be helpful for treatment in the future.

However, our research has some limitations. First, our data are from TCGA, and all of the analyses are based on this, which may lead to bias. If we perform a comprehensive analysis of data from other sources, we may obtain different results. Second, we did not conduct experiments to validate the differences in the levels of molecular transcription and expression, which undoubtedly reduces its credibility. Finally, we lack clinical follow-up data to prove the value of our prognostic model.

## Conclusion

In this article, we systematically evaluated the value of necroptosis-related lncRNAs in predicting survival, the role of the tumor microenvironment and immune cell infiltration, the potential regulatory mechanism of lncRNAs associated with necroptosis, and the prediction of potential compounds for the treatment of CC. Six lncRNA features associated with necroptosis could predict the survival of patients with CC and may be helpful for individualized treatment of cancer patients in the future.

## Data Availability

The original contributions presented in the study are included in the article/Supplementary Material. Further inquiries can be directed to the corresponding authors.

## Supplementary Material

Supplementary Figure S1 The workflow of the current study.

Supplementary Figure S2
**(A)** Consensus clustering distribution function (CDF) for k = 2 to 9. **(B)** Area under CDF curve increment for k = 2 to 9. **(C)** Tracking plot for k = 2 to 9. **(D)** LASSO coefficient profiles of the necroptosis-related lncRNAs. **(E)** Partial likelihood deviance of different numbers of variables revealed by the LASSO regression model.

Supplementary Figure S3 Analysis of the difference of immune cells and gene correlation analysis in three clusters. **(A)** B cells memory. **(B)** B cells naive. **(C)** Dendritic cells activated. **(D)** Dendritic cells resting. **(E)** Eosinophils. **(F)** Macrophages M0. **(G)** Macrophages M1. **(H)** Macrophages M2. **(I)** Mast cells activated. **(J)** Mast cells resting. **(K)** Monocytes. **(L)** Neutrophils. **(M)** NK cells activated. **(N)** NK cells resting. **(O)** Plasma cells. **(P)** T cells CD4 memory activated. **(Q)** T cells CD4 memory resting. **(R)** T cells CD4 naïve. **(S)** T cells CD8. **(T)** T cells follicular helper. **(U)** T cells gamma delta. **(V)** T cells regulatory (Tregs). **(W)** Expression of CTLA-4. **(X)** Expression of PD-1. **(Y)** Expression of PD-L1.
