# Supplementary material for: Comprehensive Analysis of Necroptosis-Related Long Noncoding RNA Immune Infiltration and Prediction of Prognosis in Patients With Colon Cancer
Source: Front Mol Biosci. 2022 Feb 14;9:811269. doi: 10.3389/fmolb.2022.811269 (PMC8883231; doi:10.3389/fmolb.2022.811269)
Supplement: Supplementary file 2 [file DataSheet2.docx]

**Table S1. Necroptosis-related genes**

| **Genes** | **Description** |
| --- | --- |
| ADAM17 | ADAM metallopeptidase domain 17 |
| AXL | AXL receptor tyrosine kinase |
| BIRC2 | baculoviral IAP repeat containing 2 |
| BIRC3 | baculoviral IAP repeat containing 3 |
| BRAF | B-Raf proto-oncogene, serine/threonine kinase |
| BRD4 | bromodomain containing 4 |
| CASP8 | caspase 8 |
| CDC37 | cell division cycle 37, HSP90 cochaperone |
| CYLD | CYLD lysine 63 deubiquitinase |
| FADD | Fas associated via death domain |
| FAS | Fas (TNF receptor superfamily member 6) |
| IPMK | inositol polyphosphate multikinase |
| ITPK1 | inositol-tetrakisphosphate 1-kinase |
| MLKL | mixed lineage kinase domain like pseudokinase |
| PARP1 | Poly (ADP-ribose) polymerase 1 |
| PELI1 | pellino E3 ubiquitin protein ligase 1 |
| PGAM5 | PGAM family member 5, mitochondrial serine/threonine protein phosphatase |
| RIPK1 | receptor interacting serine/threonine kinase 1 |
| RIPK3 | receptor interacting serine/threonine kinase 3 |
| SP1 | Sp1 transcription factor |
| TICAM1 | toll like receptor adaptor molecule 1 |
| TLR3 | toll like receptor 3 |
| TLR4 | toll like receptor 4 |
| TNF | tumor necrosis factor |
| TNFAIP3 | TNF alpha induced protein 3 |
| TNFRSF1A | TNF receptor superfamily member 1A |
| TRADD | TNFRSF1A associated via death domain |
| TRAF2 | TNF receptor associated factor 2 |
| TRAF5 | TNF receptor associated factor 5 |
| UHRF1 | ubiquitin like with PHD and ring finger domains 1 |
| ZBP1 | Z-DNA binding protein 1 |

**Table S2. Prognosis related lncRNA**

| **Gene** | **HR** | **HR.95L** | **HR.95H** | **P value** |
| --- | --- | --- | --- | --- |
| ZNF674-AS1 | 1.159824 | 1.011967 | 1.329283 | 0.033094 |
| LINC00973 | 1.385495 | 1.031239 | 1.861447 | 0.030454 |
| KMT2E-AS1 | 1.114516 | 1.039781 | 1.194622 | 0.002202 |
| TNFRSF10A-AS1 | 0.880704 | 0.778817 | 0.99592 | 0.042853 |
| PDE2A-AS2 | 3.080938 | 1.133569 | 8.373708 | 0.027404 |
| LINC01679 | 3.454153 | 1.1421 | 10.4467 | 0.028144 |
| PCED1B-AS1 | 1.230713 | 1.007224 | 1.503791 | 0.04232 |
| PAN3-AS1 | 1.258207 | 1.02617 | 1.542712 | 0.02722 |
| HCG27 | 1.615249 | 1.026364 | 2.542012 | 0.038225 |
| MORF4L2-AS1 | 1.891011 | 1.014417 | 3.525103 | 0.044962 |
| GABPB1-AS1 | 1.170999 | 1.007882 | 1.360515 | 0.039156 |
| MIR3936HG | 1.570273 | 1.078759 | 2.285735 | 0.018486 |
| MACORIS | 3.458709 | 1.317815 | 9.077653 | 0.011718 |
| OSGEPL1-AS1 | 2.216519 | 1.146632 | 4.284685 | 0.01794 |
| CCDC28A-AS1 | 5.252177 | 1.169059 | 23.59621 | 0.030485 |
| MALINC1 | 3.532372 | 1.528241 | 8.164714 | 0.003156 |
| PRKAR1B-AS2 | 2.181585 | 1.347891 | 3.530932 | 0.001498 |
| C1orf220 | 2.455667 | 1.094165 | 5.511326 | 0.029395 |
| PRR7-AS1 | 1.418632 | 1.014885 | 1.983 | 0.040714 |
| FAM66C | 3.741657 | 1.071746 | 13.06279 | 0.038585 |
| NCBP2-AS1 | 1.567921 | 1.073658 | 2.289721 | 0.019922 |
| SNHG7 | 1.038856 | 1.006955 | 1.071767 | 0.016596 |
| ATP2B1-AS1 | 8.408046 | 2.616827 | 27.01563 | 0.00035 |
| NCK1-DT | 1.284741 | 1.045467 | 1.578777 | 0.017181 |
| LINC01138 | 1.539569 | 1.100446 | 2.153921 | 0.011781 |
| WARS2-AS1 | 1.370438 | 1.056073 | 1.778383 | 0.017772 |
| LINC01215 | 1.874437 | 1.00744 | 3.487565 | 0.047327 |
| LINC01857 | 1.427881 | 1.156707 | 1.762628 | 0.000917 |
| SNHG26 | 1.470605 | 1.058778 | 2.042617 | 0.02141 |
| LINC02381 | 1.21445 | 1.033895 | 1.426536 | 0.017989 |
| NSMCE1-DT | 18.05743 | 3.994382 | 81.63234 | 0.000171 |
| CAPN10-DT | 1.862035 | 1.209749 | 2.866027 | 0.004723 |
| PAXIP1-AS2 | 1.698038 | 1.098918 | 2.623795 | 0.017088 |
| FALEC | 3.685396 | 1.991516 | 6.820004 | 3.27E-05 |
| ZEB1-AS1 | 2.328768 | 1.628011 | 3.331157 | 3.69E-06 |
| ZKSCAN2-DT | 1.481083 | 1.267875 | 1.730144 | 7.31E-07 |
| LCMT1-AS1 | 27.06852 | 6.7262 | 108.933 | 3.43E-06 |
| LENG8-AS1 | 1.140351 | 1.024377 | 1.269454 | 0.016391 |
| SEPTIN7-DT | 4.673139 | 1.775334 | 12.30092 | 0.001794 |
| TMED2-DT | 2.972855 | 1.245198 | 7.097562 | 0.014133 |
| SMG7-AS1 | 4.347513 | 1.017926 | 18.56802 | 0.047262 |
| DGUOK-AS1 | 1.180029 | 1.017406 | 1.368647 | 0.028666 |
| ARRDC1-AS1 | 1.211371 | 1.080233 | 1.358429 | 0.001037 |
| ASH1L-AS1 | 1.360403 | 1.085262 | 1.7053 | 0.007592 |
| LINC00861 | 1.53901 | 1.086588 | 2.179806 | 0.015202 |
| MYOSLID | 5.084372 | 1.412267 | 18.3045 | 0.012842 |
| DUXAP8 | 2.002872 | 1.177536 | 3.406689 | 0.010377 |
| MIR600HG | 1.447552 | 1.067755 | 1.962442 | 0.01721 |
| PHC2-AS1 | 2.027947 | 1.073892 | 3.829593 | 0.029276 |
| LINC01480 | 3.013531 | 1.668511 | 5.442797 | 0.000255 |
| LINC01106 | 1.519032 | 1.126121 | 2.049032 | 0.006185 |
| LINC01237 | 3.112003 | 1.656173 | 5.847553 | 0.000419 |
| LINC01503 | 1.245139 | 1.057958 | 1.465437 | 0.008344 |
| ITGB1-DT | 2.323377 | 1.369581 | 3.941412 | 0.00177 |
| LINC02175 | 3.102178 | 1.299984 | 7.402788 | 0.010736 |
| STAM-AS1 | 4.060734 | 1.326847 | 12.42763 | 0.014069 |

**Table S3. LncRNA related to prognostic model**

| **Gene** | **Coef** |
| --- | --- |
| MACORIS | 0.142491 |
| FALEC | 0.279156 |
| ZEB1-AS1 | 0.195714 |
| ZKSCAN2-DT | 0.031056 |
| LCMT1-AS1 | 0.45283 |
| MYOSLID | 0.446685 |
